# Human Papillomavirus Epidemiology and Prevention: Is There Still a Gender Gap?

**DOI:** 10.3390/vaccines11061060

**Published:** 2023-06-04

**Authors:** Giovanna Milano, Giovanni Guarducci, Nicola Nante, Emanuele Montomoli, Ilaria Manini

**Affiliations:** 1 Department of Life Sciences, University of Siena, 53100 Siena, Italy; giovanna.milano@outlook.com; 2 Post Graduate School of Public Health, University of Siena, 53100 Siena, Italy; giovanni.guarducc@student.unisi.it (G.G.); nicola.nante@unisi.it (N.N.); 3 Department of Molecular and Developmental Medicine, University of Siena, 53100 Siena, Italy; montomoli@unisi.it; 4 VisMederi S.r.l., 53100 Siena, Italy; 5 Interuniversity Research Centre on Influenza and Other Transmissible Infections (CIRI-IT), 16132 Genoa, Italy

**Keywords:** human papillomavirus, epidemiology, prevention strategies, vaccination, screening, cervical cancer, gender gap

## Abstract

Background and aim: Human papillomavirus (HPV) is sexually transmitted, one of the three most common sexually transmitted infections (STIs) in both males and females, and the most common viral STI. A crucial public health strategy to protect people against HPV is vaccination, which has shown its effectiveness in preventing HPV-related diseases. Presently, three types of vaccines are available (bivalent, quadrivalent, and nonvalent), and they all target the two most oncogenic virus genotypes (HPV 16 and 18). In recent years, the need to implement vaccination programmes that include all genders has been discussed in order to achieve herd immunity against HPV. To date, only a few countries have included young males in their vaccination programmes. Thus, our objective with this review is to provide an overview of the epidemiology of HPV and HPV prevention strategies and report the latest findings from the scientific literature.

## 1. Introduction

Human papillomavirus (HPV) infections are the most common sexually transmitted infections worldwide [1]. They are prevalent in young women, but infections may also occur in sexually active adults [2].

HPVs are icosahedral viruses with a double-stranded DNA genome belonging to the Papillomaviridae family, which consists of 39 genera [3,4]. In turn, HPVs are divided into five genera (alpha, beta, gamma, mu, and nu) based on the L1 protein; these genera contain more than 200 types distributed differently across geographic areas [5]. The Alpha papillomavirus genus is, in turn, divided into two categories based on its power to develop benign or malignant tumors [6]. HPVs infect basal epithelial cells, which are the under-differentiated, deep-layer cells of the skin and/or mucous membranes [1]. The viral genome is characterized by eight ORFs (open reading frames) divided into seven early (E) and two late (L) genes. The L region encodes the two viral capsid proteins. The oncogenic E5, E6, and E7 proteins encoded by the high-risk types can transform and stimulate cell growth in the basal and parabasal layers [5,6]. The E6 and E7 genes inhibit tumor suppressors such as p53 and pRb, which regulate the cell cycle and apoptosis, respectively, leading to an elevated risk of cancer development [7].

Globally, 4.5% of all cancers are attributable to HPVs, which are responsible for 8.6% of cancer cases in women (the third most prevalent cause, with a high mortality) and 0.8% in men [8]. The low-risk genotypes are usually associated with genital warts and respiratory tract papilloma, while the high-risk genotypes are associated with a malignant transformation of cells, as in oropharyngeal and anogenital cancers [3]. The associations between high-risk (HR) HPVs and some types of cancer are well-established [2,9], not only with the most common HR-HPV types 16 and 18 but also with the less-prevalent types 31, 33, 45, 52, and 58 [8]. Types 16 and 18 are strongly associated with cervical and penile cancers, as well as with anal and oropharyngeal cancers [3,10,11].

Most HR-HPV infections do not develop into external lesions and remain asymptomatic, and they are eventually immunologically cleared [12,13]. The first vaccine against HPV has been available since 2006. To date, there are three types of vaccines available against HPVs, and they have been progressively introduced into many national vaccination programs. Unfortunately, however, several studies and international agencies have reported that both the introduction of the vaccine and the coverage achieved are still suboptimal [14,15]. The purpose of this overview is to summarize recent studies in order to highlight how epidemiology and prevention strategies have developed in recent years.

## 2. Global Epidemiology of HPV and Its Related Diseases

HPV infections and related diseases affect both women and men. In fact, it is estimated that about 80% of sexually active women and men will be infected with HPV at least once during their lifetime [16]. HPV infection is the second most common cause of cancer after gastric cancer, which is caused by *Helicobacter pylori* [17,18]. However, the epidemiology of HPV differs between male and female populations [19]. Worldwide, in the female population, 26.8% of cases afflict the genital organs (this rate is the highest in Sub-Saharan Africa) and 14% afflict the anus, whereas in the male population, 45.2% of the cases afflict the genital organs and 16% afflict the anus [20,21].

**Figure 1:**
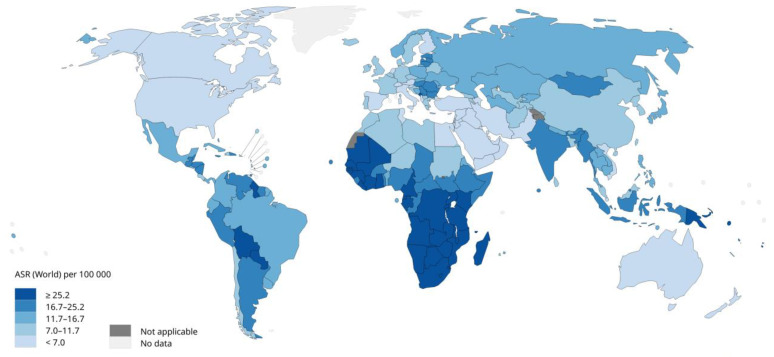
Age-standardized incidence rates of cervical cancer in the world (2020) (Source: International Agency Research on Cancer, WHO 2020). Licence: CC BY-NC-SA 3.0 IGO.

Figure 1 [22] shows the age-standardized incidence rates of cervical cancer, for which HPV is responsible for 97% of all cases. HPV-16 and 18 cause 90% of HPV-related cancers [14], although cases linked to HPV 45, 33, and 35 have been increasing [23,24]. Figure 1 [22] also demonstrates the high incidence of cervical cancer in many African nations as well as some Southeast Asian and Latin American nations. In women, persistent infection increases the risk of developing intraepithelial lesions or cervical cancer, which represents about 80% of HPV-related cancers. Usually, the prevalence of cervical infection decreases after the age of 30 [14,25]. It has also been observed that cervical infection results in a higher prevalence of HPV-related anal diseases [25]. Seventy percent of vulvar cancer and 75% of vaginal cancer cases are HPV-related [26].

Regarding HPV infections among men, the rates are higher in men who are sexually active with other men and men who are HIV-positive [27]. Globally, HPVs are responsible for 50.8% cases of penile cancer, 79.8% of penile intraepithelial neoplasia (PeIN), and 90% of genital warts. Furthermore, HPV 16 is the most common cause of these lesions, with a pooled prevalence of 68.3% of penile cancer cases and 69.8% of PeIN cases [18].

Additionally, HPVs cause around 26–30% of head–neck cancers [25]. At present, the incidences of HPV-associated (up to 90% of HPV-16-positive cases) oropharyngeal squamous cell carcinoma (OPSCC) cases are increasing in developed countries [27,28]. Globally, 33% of HPV+ OPSCC cases were reported in 2021; however, the prevalence varies considerably depending on the country, with estimates ranging from 0% in southern India to 85% in Lebanon [29]. In addition, HPV+ cases of OPSCC are more prevalent than HPV– cases of OPSCC in people who do not consume tobacco or alcohol [30]. The prevalence of HPV+ OPSCC was previously reported to decrease with increasing age, and historically, most cases of HPV+ OPSCC occur in men. [30,31]. The incidence of HPV+ OPSCC has greatly increased over the last two decades in some European countries [30,32,33], while in lower-middle-income South Asia and sub-Saharan Africa, epidemiological reports are scarce, so it is consequently unclear whether similar upward trends are absent or simply undetected in these regions [34]. A 26% incidence of HPV is found in both conjunctival intraepithelial neoplasia and in conjunctival squamous cell carcinoma (with high rates of genotypes 16, 18, and 33); HPV infection causes an eightfold increase in the likelihood of developing these two neoplasms [35].

## 3. Transmission

Worldwide, the best-known route of HPV transmission is sexual transmission, which principally causes the high incidence of HPV in anogenital warts and cervical cancer. However, HPVs are also diagnosed in children and adolescents who are not sexually exposed, so other means of transmission are considered in studies.

HPV has been found in infants’ mouths, breast milk, amniotic fluid, the placenta, umbilical blood, spermatozoa, and seminal fluid [36]. However, since the papillomavirus has historically been associated with intercourse, the increasing incidence of anogenital warts in infants would lead one to suspect sexual violence. Fortunately, this hypothesis has been abandoned [13]. The virus can be transmitted from the mother to the embryo, fetus, or child during pregnancy or delivery, although the mechanism is still not understood. A study by Mastora et al. showed that mouse sperm can internalize HR-HPV genes [37]. Therefore, the infection can also occur at the time of fertilization [12]. The presence of the same HPV type in mothers and newborn children suggests perinatal transmission [38].

HPV-related warts have also been found people who have never had sexual intercourse; therefore, several routes of horizontal HPV transmission have been highlighted [13], which are autoinoculation, hetero-inoculation, or via fomites. At the genital level, an HPV infection not transmitted through sexual acts can be spread via contact between infected fingers and genitals. For children, this incidence is associated with a parent or caretaker who has hand warts and could transmit an infection to the infant while changing diapers and cleaning the anogenital area [12].

We also know that there is a risk of transmission through endovaginal ultrasound probes that have been inadequately disinfected and are therefore ineffective at preventing HR-HPV contamination. This represents a risk of nosocomial HR-HPV transmission during an ultrasound procedure [39]. In a different set of circumstances, the HPV virus appears to be transmitted through surgical smoke generated during laser ablation procedures. Thus far, this risk of infection has been documented in animal models: bovine papillomavirus collected from CO_2_ laser smoke during wart treatment in cattle induced cutaneous fibropapillomas when re-inoculated into the skin of calves. However, this risk of infection remains controversial in humans [40,41,42].

Lastly, the waterborne transmission of HPV has never been proven, even though we know that HPV 16 remains infectious for seven days on wet surfaces [43,44,45], and HPV samples have been detected in raw sewage and sewage sludge [46,47,48,49]. HPV was detected in 50% of samples of domestic bathing water [50].

## 4. Clinical Manifestation

### 4.1. Benign

Anogenital warts and recurrent respiratory papillomatosis are mainly caused by HPV 6 and 11. Almost all anogenital warts (90%) are caused by low-risk HPVs which do not have oncogenic potential, even if occasional high-risk types are detected as co-infections with HPV 6 and 11 [51]. Although most anogenital warts are considered benign lesions, in some cases, they are associated with an increased risk of developing grade 2 or worse cervical intraepithelial neoplasia (CIN), intraepithelial neoplasia, and anogenital cancer [52].

Recurrent respiratory papillomatosis (RRP) is usually a benign condition affecting the upper aerodigestive tract following HPV-6 and HPV-11 infection. Two forms are recognized and distinguished according to the age of the onset of symptoms: JoRRP (juvenile-onset RRP) and AoRRP (adult-onset RRP). The former, which is probably related to perinatal infections, is very frequent, especially in sub-Saharan Africa, and is present in individuals younger than 12 years of age. The adult form, which is frequent in Europe and South America, is generally related to the practice of oral sex. Papillomatosis is the most prevalent in the larynx but sometimes involves the trachea, oropharynx, nasopharynx, nose, oral cavity, and lung parenchyma. Its clinical manifestation is characterized by a progressive hoarseness that may evolve into a “cauliflower-like” lesion, which could potentially develop to a point of obstructing the airway, consequently requiring clinical intervention and sometimes even tracheostomy [53,54].

Cutaneous warts (CWs), also known as verrucae, generally affect children and young adults and peak between 10 and 14 years of age. CWs are mainly caused by the genera beta and gamma (HPV 4 and 65) and rarely by the other strains. HPVs are detected in the commensal cutaneous flora of healthy individuals but can evolve into CWs due to viral multiplication. Most lesions, however, resolve with no treatment within two years [13]. Infections occur through skin-to-skin contact with infected individuals (often without clinical manifestations) and could possibly be transmitted from inanimate objects [55]. CW infection is higher in males than females, in young people, and in immunocompromised individuals. Individuals who are immunocompromised could later develop squamous cell carcinoma likely related to latent HPV reactivation [56].

HPV 6 and 11 are also the major culprits of condyloma acuminata (CA), which are warts located in the anogenital region or on the tongue and lips. CA occurs in both sexes with a symptomatology that varies from asymptomatic to painful. Their morphology is likewise variable: they can be flat or take on a cauliflower-like shape [57,58]. In some cases, they resolve spontaneously, but others require removal via an immunomodulatory approach or physical intervention. In some cases, surgery is recommended to prevent the progression to cancer. When the location of CA is intravaginal or intra anal, surgery is the only form of intervention available [57].

### 4.2. Malignant

The persistence of HR-HPV genotypes, in addition to other common risk factors such as cigarette smoking, the use of oral contraceptives, consuming alcohol, etc., can result in non-silent mutations in onco-suppressor genes (such as p 53) and/or proto-oncogenes that lead to the over-proliferation of cells and initiate the carcinogenesis process. It has been observed that the triggering event for the development of HPV-related malignant lesions is the integration of viral oncoproteins E5, E6, and E7 within the human genome. Genotypes 16 and 18 are predominantly responsible for carcinomas of the cervix, the anogenital area (vulva, vagina, penis, and anus) and the head and neck (mouth, tonsils, pharynx, and larynx) [36,59]. The most clinically encountered cervical cancers (CC) are squamous cell carcinoma (SCC) and adenocarcinoma (AC), with a higher incidence of the former than the latter, while the most common histological types of cervical cancer are keratinized squamous cell carcinoma and non-keratinized squamous cell carcinoma, adenocarcinoma, and adenosquamous carcinoma [60,61].

In a low percentage of women, permanent HR-HPV, in conjunction with other risk factors, can induce low-grade squamous intraepithelial lesions (L- SIL), which include a mild dysplasia known as CIN 1 (cervical intraepithelial neoplasia grade 1), which can then evolve into high-grade squamous intraepithelial lesions (H- SIL). H- SIL is a progressive lesion that evolves into moderate (CIN 2) and then severe dysplasia (CIN 3). Epithelial involvement transitions from being minimal and limited in CIN 1 (involving less than one-third of the epithelium) to the involvement of two-thirds of the epithelium in CIN 2 to complete epithelial involvement in CIN 3. When the entire thickness of the epithelium of the cervix is involved, it can be defined as in situ cervical carcinoma (ISCC) [60]. A correlation between the vaginal microbiome and tumor progression has also been observed in some studies. Specifically, it appears that the severity of cervical lesions is promoted by a decrease in lactobacilli that can be observed following infection with high-risk papilloma genotypes [62].

Penile cancer is an aggressive disease which, like all cancers, implicates several risk factors, including phimosis, lichen sclerosis, inflammatory conditions (balanitis xerotica obliterans), premalignant lesions (Bowen’s disease and erythroplasia of Queyrat), high numbers of sexual partners, socioeconomic status, and HPV infections (especially with high-risk genotypes such as 16 and 18). The papillomavirus is found most often in basaloid squamous carcinoma, warty carcinoma, clean cell carcinoma, and lymphoepithelioma-like carcinoma [10,63]. The overexpression or downexpression of miRNAs have diagnostic and prognostic value. The downexpression of both hsa- miR-218 and miRNA- 246a and the overexpression of EFGR have been observed in HPV-related penile cancers. In the case of penile cancer, clinical intervention is a total or partial penectomy; therefore, precancerous lesions, when identified, should be treated immediately [64].

Head and neck squamous cell carcinomas (HNSCCs) are tumors arising from the mucosal epithelia of the oral cavity, pharynx, and larynx. High incidences are mainly determined by personal habits, but the involvement of HR-HPV is increasingly considered. HNSCCs are therefore distinguished into HPV-related HNSCCs and HPV-unrelated HNSCCs (HPV-negative HNSCCs), with the former generally having a better prognosis than the latter [65]. HPV + HNSCCs usually occur following infection in the deep crypts of the palatine and lingual tonsils and demonstrate different gene expressions, mutations, and immune profiles than HPV-negative HNSCC, which is most related to the use of tobacco or alcohol [65,66].

## 5. Prevention

### 5.1. Primary Prevention

Although HPV infections usually regress spontaneously within 1 to 2 years and not all genotypes are implicated in cancer [9,43,67,68,69], HPV infections and related diseases should be taken seriously. To date, vaccines provide the best and most cost-effective option for prevention. All vaccines currently in use are based on virus-like particles (VLPs) of the L1 protein, which is considered a strong immunogen protein that spontaneously self-assembles from pentamers. [13,36,70,71]. Gardasil (4vHPV) was the first vaccine to be approved (in 2006) by the Food and Drug Administration. It targets four types of HPV (6, 11, 16, and 18) [3]. The bivalent vaccine Cervarix (2vHPV), which targets HPV 16 and 18, was approved in 2007. It has been proven that 2vHPV also protects against HPV 31, 33, and 45 and reduces genital warts caused by HPV 6 and 11 [3,43]. Both vaccines contain different adjuvants; the proteins of Cervarix are produced in baculovirus-infected insect cells, while Gardasil’s proteins are produced in *Saccharomyces cerevisiae* [69]. Gardasil later developed a nine-valent (9vHPV) version which further includes five L1 VLPs, particularly the oncogenic HPV types 31, 33, 45, 52, and 58, and when compared to 4vHPV, it demonstrates the best rates of prevention of low- and high-risk human papillomavirus infections. Approved by the FDA (Food and Drug Administration) in 2014, it is considered the best way to prevent CINs I, II, and III and 90% of HPV- related diseases compared to its predecessors [3,72], and in 2020, it received an indication for the prevention of some head and neck cancers caused by HPV [69].

Specifically, significant decreases in HPV 6/11/16/18 were observed in vaccinated women compared to unvaccinated women, demonstrating the high efficacy of the vaccine. In addition, there was a significant drop in incidences of HPV 6/11/16/18 in unvaccinated women as a result of herd protection. For males, the quadrivalent HPV vaccine may reduce the incidence of external genital lesions, including condyloma acuminate, grades I and II of anal intraepithelial neoplasia, and persistent HPV 6/11/16/18 infections. Finally, HPV vaccination showed high efficacy against oral HPV 16/18 infections, and a significant proportion of participants developed IgG antibodies in their oral fluid after vaccination [73].

According to recommendations from the CDC (Centers for Disease Control and Prevention) and ACIP (Advisory Committee on Immunization Practices), two or three doses of 9vHPV are recommended for both males and females ages 9 to 45 years. [4,72]. To ensure greater access to vaccination in countries with low vaccination coverage and especially in low- and middle-income countries, administering a single dose of the bivalent or nine-valent vaccine could be a viable strategy to ensure the uniformity of vaccination coverage. In fact, several studies have shown how the administration of a single dose of an HPV vaccine results in the appearance of neutralizing antibodies which, although present at a lower titer when compared to those that appear following two or three dose schedules, are able to prevent infection with the virus and prevent the appearance of precancerous lesions at the level of the cervix. Moreover, immunity also appears to be mediated by non-neutralizing antibodies and neutrophil degranulation, and the avidity indexes of antibodies directed toward the L1 protein are very similar to those of individuals receiving three doses [74,75]. Both the Gardasil and Cervarix vaccines induce cross-protection against nonvaccine types such as HPV 31 and HPV 45 but with lower antibody titers than the epitopes included in the vaccines [76,77]. Vaccination can be provided from 9 years of age; however since 2006, catch-up vaccinations have been recommended for females up to the age of 26, and catch-up vaccinations have been recommended for males up to the age of 21 since 2011, in addition to some special populations up to the age of 26. Furthermore, since the incidence of HPV infection in adults is increasing, vaccination is currently recommended for women and men aged 45 years [2].

**Figure 2:**
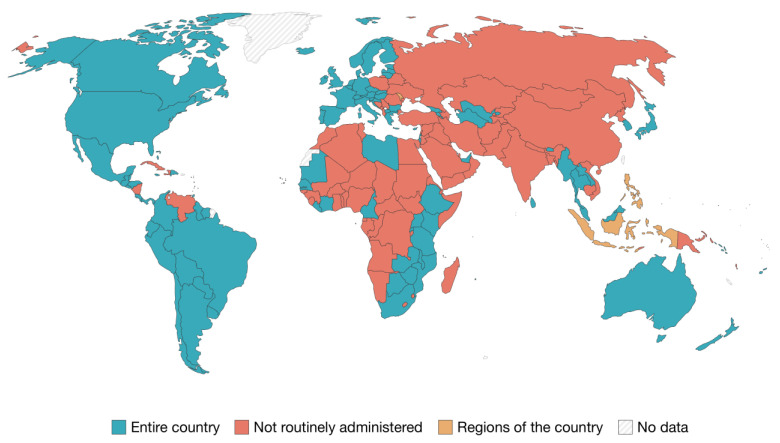
Countries which provide and recommend HPV vaccines through routine services (2021). Source: OurWorldInData.org/vaccination. Licence: CC-BY.

Figure 2 [78] shows which countries provide and recommend HPV vaccines through routine services. Australia was one of the first countries to introduce a publicly funded national HPV vaccination program in 2007. This not only resulted in a 70% decrease in the incidence of HPV 6, 11, 16 and 18 infections but also achieved high vaccination coverage for both gender groups [24,36,69,79]. Primary prevention against HPV is available due to the presence of vaccines targeting high-risk genotypes; in fact, since 2006, more than 110 countries have implemented vaccination campaigns against HPV, but only about 40 countries have introduced programs that are gender-neutral; Australia and the United States were among the first to take this step in 2011 and 2013, respectively [63,65,80].

**Figure 3:**
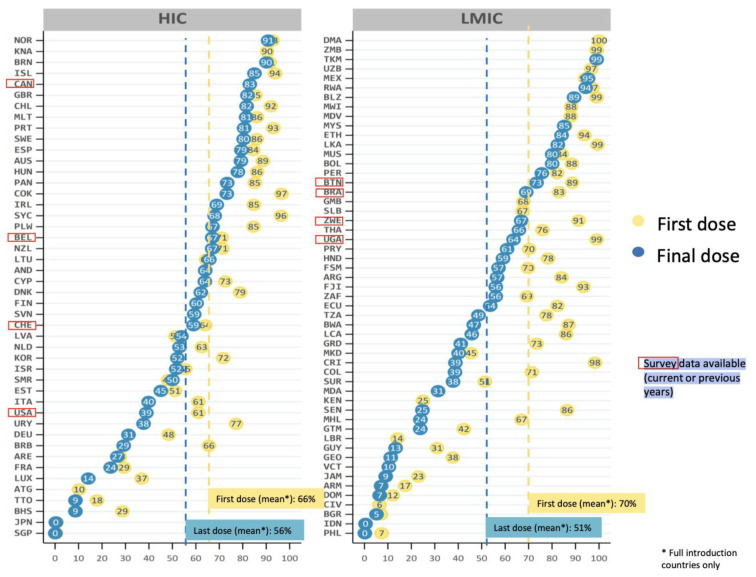
Percentages of HPV vaccine coverage globally, stratified by high and low-middle income countries. Source: Progress and Challenges with Achieving Universal Immunization Coverage—2019 WHO/UNICEF Estimates of National Immunization Coverage, World Health Organization 2020). Licence: CC BY-NC-SA 3.0 IGO.

Figure 3 shows the global HPV vaccine coverage (%) divided by income strata [81]. Global vaccination coverage is at about 12–15%, with 15% of girls and 4% of boys having received the full course of the HPV vaccine, and 20% of girls and 5% of boys having received a single dose [82]. HPV vaccination coverage varies substantially across countries, with about half of low-middle-income countries and one third of high-income countries achieving a vaccination coverage of about 80% with the first HPV vaccine dose [24,81].

Herd immunity is impossible to achieve if males are not included in vaccination programs. In fact, adopting gender-neutral HPV vaccination schedules will reduce population-transmitted infections, combat misinformation, minimize vaccine-related stigma, and promote gender equity [83,84]. To date, only a few countries have achieved a vaccination coverage of 70% [84], with a marked discrepancy between continents ranging from 20% in sub-Saharan Africa to 77% in New Zealand. In a few African countries, the bivalent and quadrivalent vaccines were made available free of charge (only for females) several years after the first vaccine was approved [85]. In Africa, it is also not easy to assess the vaccine’s efficacy on HPV-attributable cancers. In fact, cost-effectiveness models are based on male or female vaccination and not on both. Moreover, economic evaluations of HPV vaccination in Africa are very limited; current recommendations for HPV vaccination for women only are based on studies conducted in other regions with different distributions of HPV infection and associated cancers. Cost–benefit models often underestimate the added value of male vaccination because they assume that female vaccination coverage is higher [86]. As in Africa, vaccination coverage is also very low in several Asian countries. In some countries, such as China, coverage is not very clear. In addition, the vaccine is not approved for use in males, it is not included in the national vaccination program, and vaccination is at one’s own expense [87]. In Japan, female coverage reached 70%; however, following reports of adverse events, which were widely covered in the media and turned out to be unrelated to vaccination, vaccination coverage fell to less than 1% and has remained this low to date [88]. In 2021, the Southeast Asian country of Bhutan was the first country to adopt a gender-neutral vaccination strategy [83,84]. In the United States, vaccination has been recommended for females since 2006 and for males since 2011. Furthermore, since 2016, only 9vHPV has been distributed [2]. In South America, data on vaccination coverage are not available for all countries; Mexico is the only country that has reached the target of 90% of females fully vaccinated by the age of 15. Moreover, not all countries have introduced the vaccine as a public health policy [89]. In Europe, coverage varies between about 14% and 86%, with the highest values in the UK and the lowest values in Bulgaria. The availability of vaccines and vaccination policies vary from country to country [90,91,92]. In Italy, the national vaccine program provides universal vaccination for the target populations, utilizing the nine-valent vaccine for both males and females from the age of 11 [93]. Poland, instead, requires citizens to pay for the HPV vaccine, and in France, national health insurance reimburses 65% of the cost [92].

Lastly, some evidence shows that male circumcision reduces the risk of certain sexually transmitted viral infections in men and consequently for their female partners. Specifically, male circumcision is associated with slight reductions in high-risk HPV, whereas licensed HPV vaccines only protect with against a limited number of HPV types with a high level of efficacy. It is therefore likely that the two interventions have important synergistic effects [94].

### 5.2. Secondary Prevention

Primary prevention is not a substitute for secondary prevention. Indeed, women should adhere to national screening programs to prevent cervical cancer [67]. The WHO recommends cervical cancer screening from the age of 30 onwards [95]. Unequal access to screening is a major reason for the dramatic disparities in cervical cancer incidence and mortality between low- and high-income countries. In 2020, in order to reduce the circulation of HPV and eliminate cervical cancer, the WHO launched the Global Strategy to Accelerate the Elimination of Cervical Cancer as a Public Health Problem initiative, to be achieved by 2030, which includes intensifying vaccination, screening, and the treatment of women with cervical cancer [80,95]. Eliminating the incidence of cervical cancer does not mean eliminating the circulation of HPV, so gender-based primary prevention is the best weapon to invest in [83].

There are many methods of assessing cancer progression, such as VIA (visual inspection with acetic acid), a procedure used to identify CIN through a solution of acetic acid with a concentration ranging from 3 to 5 percent. Interpretation of the results is crucial in determining positivity and negativity, as well as the path of clinical treatment chosen in the case of positivity. Since 1990, some countries have used EIA as the primary screening method [96]. To assess the squamocolumnar junction, which is the area that has the highest risk of developing dysplasia, a Papanicolau test (Pap test) or the thin-layer liquid-based cytology method are performed [97]. Colposcopy is a diagnostic procedure used to examine the cervix, vagina, and vulva of women who have previously undergone VIA or who have tested positive for Papanicolau (Pap) or HPV DNA testing for high-risk oncogenic genotypes. It is also a test used after the treatment of intraepithelial and invasive carcinoma [98]. Viral genome identification can be carried out via the HPV DNA test, a multiplex test capable of detecting the high risk-HPV genome via PCR [99]. Currently, the Roche Cobas^®^ test is the only HPV test approved by the FDA as an independent screening test for women 25 years of age and older [99,100]. Genome amplification should not involve only the L1 protein, as it may be unexpressed after viral infection. A more thorough option is HPV mRNA testing the expression of oncoproteins E6 and E7, which are detectable markers of the integration of the viral genome with that of the host [99].

As cancer is a multifactorial disease, a positive HPV DNA test does not necessarily mean that one has cancer, but further investigation with colposcopy is necessary. Moreover, in some cases, HPV DNA testing may be negative in women with high-grade precancer due to a low viral load [100]. An additional marker of HPV oncogenic activity is the tumor suppressor protein p16, which is overexpressed following the demethylation of its promoter at oncoprotein E7 [101]. The use of immunohistochemical techniques to mark p16 could reduce overtreatment and conization, which could result in preterm delivery [101,102]. In addition, a minimally invasive, safe, and emotionally comfortable practice is vaginal self-sampling, which is also screening test with high sensitivity and specificity (both greater than 90%). Although it is recommended by the WHO and the International Agency for Research on Cancer as a form of screening for HPV and would provide information about the epidemiological status of under-screened countries, this practice is not widely used globally, with only 35% of countries having introduced self-sampling into their national screening programs [103,104].

In men and for other HPV+ cancers, there is still no validated method of diagnosis or screening; however, the papillomavirus has been detected in several samples: seminal fluid, phenocopy with acetic acid, urethral specimens, and penile scrapings (taken from the crown of the glans of the penis, from the inner layer of the foreskin, and from the body of the penis). Among the detection methods, polymerase chain reaction (PCR) tests are the most widely used [105,106,107,108,109].

### 5.3. Tertiary Prevention

Tertiary prevention targets both the clinical and outcome phases of a disease. It is implemented in symptomatic patients and aims to reduce the severity of the disease and any associated sequelae [110]. For HPV-induced cancers, some retrospective studies show a significant protective effect of the HPV vaccine in women and men surgically treated for HPV disease [111,112,113]. Indeed, in males who have sex with males, adjuvant vaccination and post treatment of high-grade squamous intraepithelial lesions is potentially the most effective approach to reducing the incidence of invasive anal cancer. Specifically, vaccination should be considered for all HIV-infected individuals aged 27 years or older who are undergoing treatment for high-grade squamous intraepithelial lesions [112]. In women, on the other hand, adjuvant vaccination reduces the risk of recurrence by about 80% for individuals treated surgically for CIN II lesions and FIGO stage 1A1 cervical cancer [113]. However, it is still under discussion whether vaccination is more effective first as a neo-adjuvant or as an adjuvant to conization, although it would appear to be within 30 days of standard treatment. [114,115]. Moreover, the reduction in recurrences after treatment in vaccinated patients was also confirmed for benign lesions [116,117]. In fact, for respiratory papillomatosis, the use of HPV vaccination as an adjunctive therapy in combination with surgery has shown to provide substantial benefits [118]. In contrast, vaccination does not seem to provide a significant secondary benefit in patients with previous ano-genital warts; however, this cannot be generalized because the trials were few and randomized [119]. Several methods have been studied for the tertiary prevention of oropharyngeal squamous cell carcinoma, although they have not yet been prospectively validated through a rigorous study [120]. These methods include oral fluid biomarkers and blood biomarkers [120]. In the former, the presence of HR-HPV DNA seems to be correlated with a risk of recurrence [121], while for blood biomarkers, it has been observed that higher levels of the E6 antibody are associated with an increased risk of recurrence [122]. Furthermore, the role of circulating HPV DNA will need further investigation. It has already been observed that in more advanced stages of disease, higher levels of circulating DNA are present [123]. Whether the level of circulating DNA is indicative of disease persistence or recurrence, as in other virus-induced carcinomas, still needs to be studied [124,125].

## 6. Conclusions

The epidemiology of HPV is heterogeneous between the sexes, with a higher prevalence of infection in ano-genital areas among males, which is even higher in some subgroups. To date, the only way to block the chain of transmission is vaccination, but HPV vaccination programs, especially gender-neutral ones, are still in their infancy in many countries, particularly in less-developed countries [83]. This makes it difficult to achieve herd immunity, especially in males who were only invited to get vaccinated years after the first vaccine was introduced. There is growing evidence that only gender-neutral vaccination will lead to substantial control of HPV-related diseases in both women and men and maximize cervical cancer prevention, especially if the vaccination coverage for girls in a particular region is not high [126]. Given the current situation, it is logical to assume that cervical cancer prevention will still rely on secondary methods of prevention in the years to come. Furthermore, another global public health goal should be to provide scientific evidence to establish optimal vaccination timing in order to prevent the greatest number of cancer recurrences and improve the outcomes of the proposed treatments. Lastly, health communication should also play a key role. In fact, standardizing both the quality and the quantity of information could lead to an increase in adherence to the various vaccination awareness campaigns, which already must overcome the prejudices and psychological factors that make promotion and prevention interventions complex. Surely, investing in promotion campaigns, as in the case of polio, for example, would both improve the cost–benefit balance (making it even more favorable than the balance achieved by primary prevention activities alone) and equalize vaccination coverage between the two genders, which still show a substantial gap [127,128].

## References

[1] Nøhr B., Kjaer S.K., Soylu L., Jensen A. (2019). High-risk human papillomavirus infection in female and subsequent risk of infertility: A population-based cohort study. Fertil. Steril..

[2] Meites E., Szilagyi P.G., Chesson H.W., Unger E.R., Romero J.R., Markowitz L.E. (2019). Human Papillomavirus Vaccination for Adults: Updated Recommendations of the Advisory Committee on Immunization Practices. MMWR Morb. Mortal. Wkly. Rep..

[3] Rosalik K., Tarney C., Han J. (2021). Human Papilloma Virus Vaccination. Viruses.

[4] Harden M.E., Munger K. (2017). Human papillomavirus molecular biology. Mutat. Res. Rev. Mutat. Res..

[5] Soheili M., Keyvani H., Soheili M., Nasseri S. (2021). Human papilloma virus: A review study of epidemiology, carcinogenesis, diagnostic methods, and treatment of all HPV-related cancers. Med. J. Islam. Repub. Iran..

[6] Egawa N., Doorbar J. (2017). The low-risk papillomaviruses. Virus Res..

[7] Goodman A. (2015). HPV testing as a screen for cervical cancer. BMJ.

[8] Roman B.R., Aragones A. (2021). Epidemiology and incidence of HPV-related cancers of the head and neck. J. Surg. Oncol..

[9] Ryndock E.J., Meyers C. (2014). A risk for non-sexual transmission of human papillomavirus?. Expert Rev. Anti Infect. Ther..

[10] Iorga L., Dragos Marcu R., Cristina Diaconu C., Alexandra Stanescu A.M., Pantea Stoian A., Dorel Mischianu D.L., Surcel M., Bungau S., Constantin T., Boda B.D. (2020). Penile carcinoma and HPV infection (Review). Exp. Ther. Med..

[11] Manini I., Montomoli E. (2018). Epidemiology and prevention of Human Papillomavirus. Ann Ig.

[12] Sabeena S., Bhat P., Kamath V., Arunkumar G. (2017). Possible non-sexual modes of transmission of human papilloma virus. J. Obs. Gynaecol. Res..

[13] Kombe Kombe A.J., Li B., Zahid A., Mengist H.M., Bounda G.A., Zhou Y., Jin T. (2021). Epidemiology and Burden of Human Papillomavirus and Related Diseases, Molecular Pathogenesis, and Vaccine Evaluation. Front. Public Health.

[14] Di Donato V., Caruso G., Petrillo M., Kontopantelis E., Palaia I., Perniola G., Plotti F., Angioli R., Muzii L., Benedetti Panici P. (2021). Adjuvant HPV Vaccination to Prevent Recurrent Cervical Dysplasia after Surgical Treatment: A Meta-Analysis. Vaccines.

[15] Bruni L., Saura-Lázaro A., Montoliu A., Brotons M., Alemany L., Diallo M.S., Afsar O.Z., LaMontagne D.S., Mosina L., Contreras M. (2021). HPV vaccination introduction worldwide and WHO and UNICEF estimates of national HPV immunization coverage 2010–2019. Prev. Med..

[16] Tao Y., Shao H., Zhang T., Pu J., Tang C. (2022). Factors Influencing Men’s Attitudes toward HPV Vaccination in Males Included in the Chinese National Immunization Program. Vaccines.

[17] Elst L., Albersen M. (2022). HPV Vaccination: Does It Have a Role in Preventing Penile Cancer and Other Preneoplastic Lesions?. Semin. Oncol. Nurs..

[18] de Martel C., Georges D., Bray F., Ferlay J., Clifford G.M. (2020). Global burden of cancer attributable to infections in 2018: A worldwide incidence analysis. Lancet Glob. Health.

[19] Yousefi Z., Aria H., Ghaedrahmati F., Bakhtiari T., Azizi M., Bastan R., Hosseini R., Eskandari N. (2022). An Update on Human Papilloma Virus Vaccines: History, Types, Protection, and Efficacy. Front. Immunol..

[20] Shen J., Zhou H., Liu J., Zhang Z., Fang W., Yang Y., Hong S., Xian W., Ma Y., Zhou T. (2020). Incidence and risk factors of second primary cancer after the initial primary human papillomavirus related neoplasms. MedComm.

[21] Bhatla N., Aoki D., Sharma D.N., Sankaranarayanan R. (2021). Cancer of the cervix uteri: 2021 update. Int. J. Gynaecol. Obstet..

[22] International Agency for Research on Cancer, World Health Organization Incidence, Prevalence and Mortality Rates (World) in 2020. https://gco.iarc.fr/today/online-analysis-map?v=2020&mode=population&mode_population=continents&population=900&populations=900&key=asr&sex=2&cancer=23&type=0&statistic=5&prevalence=0&population_group=0&ages_group%5B%5D=0&ages_group%5B%5D=17&nb_items=10&group_cancer=1&include_nmsc=0&include_nmsc_other=0&projection=natural-earth&color_palette=default&map_scale=quantile&map_nb_colors=5&continent=0&show_ranking=0&rotate=%255B10%252C0%255D.

[23] Santella B., Schettino M.T., Franci G., De Franciscis P., Colacurci N., Schiattarella A., Galdiero M. (2022). Microbiota and HPV: The role of viral infection on vaginal microbiota. J. Med. Virol..

[24] Calabrò G.E., Ricciardi W. (2022). Verso un Mondo HPV Free: Strategie Internazionali, da Implementare a Livello Nazionale, per L’eliminazione del Cancro Cervicale: Il Valore Della Prevenzione e Della Vaccinazione Anti-HPV Negli Adolescenti Da: I Numeri del Cancro in Italia. AIOM-AIRTUM. https://www.aiom.it/wp-content/uploads/2022/12/2022_AIOM_NDC-web.pdf.

[25] Pimple S., Mishra G. (2022). Cancer cervix: Epidemiology and disease burden. Cytojournal.

[26] Efua Sackey M., Markey K., Grealish A. (2022). Healthcare professional’s promotional strategies in improving Human papillomavirus (HPV) vaccination uptake in adolescents: A systematic review. Vaccine.

[27] Sasidharanpillai S., Ravishankar N., Kamath V., Bhat P.V., Bhatt P., Arunkumar G. (2021). Prevalence of Human Papillomavirus (HPV) DNA among Men with Oropharyngeal and Anogenital Cancers: A Systematic Review and Meta-Analysis. Asian Pac. J. Cancer Prev..

[28] Lehtinen M., Gray P., Louvanto K., Vänskä S. (2022). In 30 years, gender-neutral vaccination eradicates oncogenic human papillomavirus (HPV) types while screening eliminates HPV-associated cancers. Expert Rev. Vaccines.

[29] Carlander A.F., Jakobsen K.K., Bendtsen S.K., Garset-Zamani M., Lynggaard C.D., Jensen J.S., Buchwald C.V., Grønhøj C. (2021). A contemporary systematic review on repartition of HPV-positivity in oropharyngeal cancer worldwide. Viruses.

[30] Lechner M., Liu J., Masterson L., Fenton T.R. (2022). HPV-associated oropharyngeal cancer: Epidemiology, molecular biology and clinical management. Nat. Rev. Clin. Oncol..

[31] Gabutti G., d’Anchera E., De Motoli F., Savio M., Stefanati A. (2021). Human Papilloma Virus Vaccination: Focus on the Italian Situation. Vaccines.

[32] Zamani M., Grønhøj C., Jensen D.H., Carlander A.F., Agander T., Kiss K., von Buchwald C., Friborg J., Andersen E., Nielsen F.C. (2020). The current epidemic of HPV-associated oropharyngeal cancer: An 18-year Danish population-based study with 2,169 patients. Eur. J. Cancer.

[33] Del Mistro A., Frayle H., Menegaldo A., Favaretto N., Gori S., Nicolai P., Spinato G., Romeo S., Tirelli G., da Mosto M.C. (2020). Age-independent increasing prevalence of human papillomavirus-driven oropharyngeal carcinomas in North-East Italy. Sci. Rep..

[34] De Martel C., Plummer M., Vignat J., Franceschi S. (2017). Worldwide burden of cancer attributable to HPV by site, country and HPV type. Int. J. Cancer.

[35] Seedat R.Y. (2020). Juvenile-Onset Recurrent Respiratory Papillomatosis Diagnosis and Management—A Developing Country Review. Pediatric Health Med. Ther..

[36] Araldi R.P., Sant’Ana T.A., Módolo D.G., de Melo T.C., Spadacci-Morena D.D., de Cassia Stocco R., Cerutti J.M., de Souza E.B. (2018). The human papillomavirus (HPV)-related cancer biology: An overview. Biomed. Pharmacother..

[37] Mastora E., Kitsou C., Evangelou T., Zikopoulos A., Zagorianakou N., Georgiou I. (2021). Presence of HPV 16 and HPV 18 in Spermatozoa and Embryos of Mice. Vivo.

[38] Rombaldi R.L., Serafini E.P., Mandelli J., Zimmermann E., Losquiavo K.P. (2009). Perinatal transmission of human papilomavirus DNA. Virol J..

[39] Casalegno J.S., Le Bail Carval K., Eibach D., Valdeyron M.L., Lamblin G., Jacquemoud H., Mellier G., Lina B., Gaucherand P., Mathevet P. (2012). High risk HPV contamination of endocavity vaginal ultrasound probes: An underestimated route of nosocomial infection?. PLoS ONE.

[40] Palma S., Gnambs T., Crevenna R., Jordakieva G. (2021). Airborne human papillomavirus (HPV) transmission risk during ablation procedures: A systematic review and meta-analysis. Environ. Res..

[41] Sawchuk W.S., Weber P.J., Lowy D.R., Dzubow L.M. (1989). Infectious papillomavirus in the vapor of warts treated with carbon dioxide laser or electrocoagulation: Detection and protection. J. Am. Acad. Dermatol..

[42] Garden J.M., O’Banion M.K., Bakus A.D., Olson C. (2002). Viral disease transmitted by laser-generated plume (aerosol). Arch. Dermatol..

[43] Petca A., Borislavschi A., Zvanca M.E., Petca R.C., Sandru F., Dumitrascu M.C. (2020). Non-sexual HPV transmission and role of vaccination for a better future (Review). Exp. Ther. Med..

[44] Ding D.C., Chang Y.C., Liu H.W., Chu T.Y. (2011). Long-term persistence of human papillomavirus in environments. Gynecol. Oncol..

[45] La Rosa G., Rose J.B., Jiménez-Cisneros B., Meschke J.S., Girones R. (2016). Papillomavirus. Global Water Pathogen Project, Part 3 Viruses.

[46] Symonds E.M. (2008). Viruses in raw sewage and their potential to indicate fecal pollution in coastal environments. Grad. Sch. Theses Diss..

[47] Cantalupo P.G., Calgua B., Zhao G., Hundesa A., Wier A.D., Katz J.P., Grabe M., Hendrix R.W., Girones R., Wang D. (2011). Raw sewage harbors diverse viral populations. mBio.

[48] Bibby K., Peccia J. (2013). Identification of viral pathogen diversity in sewage sludge by metagenome analysis. Environ. Ment. Sci. Technol..

[49] Fratini M., Di Bonito P., La Rosa G. (2014). Oncogenic papillomavirus and polyomavirus in water environments: Is there a potential for waterborne transmission?. Food Environ. Virol..

[50] Di Bonito P., Della Libera S., Petricca S., Iaconelli M., Sanguinetti M., Graffeo R., Accardi L., La Rosa G. (2015). A large spectrum of alpha and beta papillomaviruses are detected in human stool samples. J. Gen. Virol..

[51] Dediol I., Buljan M., Vurnek-A Ivkoviä M., Bulat V., A Itum M., A Ubriloviä A. (2009). Psychological burden of anogenital warts. J. Eur. Acad. Dermatol. Venereol..

[52] Tyros G., Mastraftsi S., Gregoriou S., Nicolaidou E. (2021). Incidence of anogenital warts: Epidemiological risk factors and real-life impact of human papillomavirus vaccination. Int. J. STD AIDS..

[53] Fortes H.R., von Ranke F.M., Escuissato D.L., Araujo Neto C.A., Zanetti G., Hochhegger B., Souza C.A., Marchiori E. (2017). Recurrent respiratory papillomatosis: A state-of-the-art review. Respir. Med..

[54] Seedat R.Y., Dikkers F.G. (2022). Global epidemiology of HPV-associated recurrent respiratory papillomatosis and effect of vaccination. Future Virol..

[55] McLaughlin-Drubin M.E., Crum C.P., Münger K. (2011). Human papillomavirus E7 oncoprotein induces KDM6A and KDM6B histone demethylase expression and causes epigenetic reprogramming. Proc. Natl. Acad. Sci. USA.

[56] Singh R.K. (2021). Diffuse Non-Genital Cutaneous Warts. Am. J. Trop. Med. Hyg..

[57] El Moussaoui S., Fernández-Campos F., Alonso C., Limón D., Halbaut L., Garduño-Ramirez M.L., Calpena A.C., Mallandrich M. (2021). Topical Mucoadhesive Alginate-Based Hydrogel Loading Ketorolac for Pain Management after Pharmacotherapy, Ablation, or Surgical Removal in Condyloma Acuminata. Gels.

[58] Pennycook K.B., McCready T.A. (2022). Condyloma Acuminata. StatPearls.

[59] Oyervides-Muñoz M.A., Pérez-Maya A.A., Rodríguez-Gutiérrez H.F., Gómez-Macias G.S., Fajardo-Ramírez O.R., Treviño V., Barrera-Saldaña H.A., Garza-Rodríguez M.L. (2018). Understanding the HPV integration and its progression to cervical cancer. Infect. Genet. Evol..

[60] Bañuelos-Villegas E.G., Pérez-yPérez M.F., Alvarez-Salas L.M. (2021). Cervical Cancer, Papillomavirus, and miRNA Dysfunction. Front. Mol. Biosci..

[61] Merz J., Bossart M., Bamberg F., Eisenblaetter M. (2020). Revised FIGO Staging for Cervical Cancer—A New Role for MRI. Rofo.

[62] Castanheira C.P., Sallas M.L., Nunes R.A.L., Lorenzi N.P.C., Termini L. (2021). Microbiome and Cervical Cancer. Pathobiology.

[63] Schlenker B., Schneede P. (2019). The Role of Human Papilloma Virus in Penile Cancer Prevention and New Therapeutic Agents. Eur. Urol. Focus..

[64] Kuasne H., Barros-Filho M.C., Busso-Lopes A., Marchi F.A., Pinheiro M., Muñoz J.J., Scapulatempo-Neto C., Faria E.F., Guimarães G.C., Lopes A. (2017). Integrative miRNA and mRNA analysis in penile carcinomas reveals markers and pathways with potential clinical impact. Oncotarget.

[65] Johnson D.E., Burtness B., Leemans C.R., Lui V.W.Y., Bauman J.E., Grandis J.R. (2020). Head and neck squamous cell carcinoma. Nat. Rev. Dis. Prim..

[66] Chen Y.H., Chien C.Y., Huang T.L., Chiu T.J., Wang Y.M., Fang F.M., Li S.H. (2023). Low p16 Cytoplasmic Staining Predicts Poor Treatment Outcome in Patients with p16-Negative Locally Advanced Head and Neck Squamous Cell Carcinoma Receiving TPF Induction Chemotherapy. Biomedicines.

[67] Bik E.M., Bird S.W., Bustamante J.P., Leon L.E., Nieto P.A., Addae K., Alegría-Mera V., Bravo C., Bravo D., Cardenas J.P. (2019). A novel sequencing-based vaginal health assay combining self-sampling, HPV detection and genotyping, STI detection, and vaginal microbiome analysis. PLoS ONE.

[68] Eun T.J., Perkins R.B. (2020). Screening for Cervical Cancer. Med. Clin. N. Am..

[69] Markowitz L.E., Schiller J.T. (2021). Human Papillomavirus Vaccines. J. Infect. Dis..

[70] Gualano M.R., Bert F., Voglino G., Buttinelli E., D’Errico M.M., De Waure C., Di Giovanni P., Fantini M.P., Giuliani A.R., Marranzano M. (2018). Collaborating Group. Attitudes towards compulsory vaccination in Italy: Results from the NAVIDAD multicentre study. Vaccine.

[71] Yazdani Z., Rafiei A., Valadan R., Ashrafi H., Pasandi M., Kardan M. (2020). Designing a potent L1 protein-based HPV peptide vaccine: A bioinformatics approach. Comput. Biol. Chem..

[72] Soca Gallego L., Dominguez A., Parmar M. (2023). Human Papilloma Virus Vaccine. StatPearls.

[73] Kamolratanakul S., Pitisuttithum P. (2021). Human Papillomavirus Vaccine Efficacy and Effectiveness against Cancer. Vaccines.

[74] Quang C., Chung A.W., Frazer I.H., Toh Z.Q., Licciardi P.V. (2022). Single-dose HPV vaccine immunity: Is there a role for non-neutralizing antibodies?. Trends Immunol..

[75] Kreimer A.R., Sampson J.N., Porras C., Schiller J.T., Kemp T., Herrero R., Wagner S., Boland J., Schussler J., Lowy D.R. (2020). Costa Rica HPV Vaccine Trial (CVT) Group. Evaluation of Durability of a Single Dose of the Bivalent HPV Vaccine: The CVT Trial. J. Natl. Cancer Inst..

[76] Panwar K., Godi A., Cocuzza C.E., Andrews N., Southern J., Turner P., Miller E., Beddows S. (2022). Multiplex Human Papillomavirus L1L2 virus-like particle antibody binding assay. MethodsX.

[77] Godi A., Panwar K., Haque M., Cocuzza C.E., Andrews N., Southern J., Turner P., Miller E., Beddows S. (2019). Durability of the neutralizing antibody response to vaccine and non-vaccine HPV types 7 years following immunization with either Cervarix^®^ or Gardasil^®^ vaccine. Vaccine.

[78] Roser M., Ortiz-Ospina E. (2021). Which Countries Include Human Papillomavirus (HPV) Vaccines in Their Vaccination Schedules?. https://ourworldindata.org/grapher/human-papillomavirus-vaccine-immunization-schedule?country=BFA~ROU~ARM.

[79] Brotherton J.M., Malloy M., Budd A.C., Saville M., Drennan K.T., Gertig D.M. (2015). Effectiveness of less than three doses of quadrivalent human papillomavirus vaccine against cervical intraepithelial neoplasia when administered using a standard dose spacing schedule: Observational cohort of young women in Australia. Papillomavirus Res..

[80] Wang W.V., Kothari S., Khoury H., Niccolai L., Garland S.M., Sundström K., de Pouvourville G., Bonanni P., Chen Y.T., Franco. E.L. (2023). A review of data systems for assessing the impact of HPV vaccination in selected high-income countries. Expert Rev. Vaccines.

[81] (2020). WHO/UNICEF. Progress and Challenges with Achieving Universal Immunization Coverage 2020. https://cdn.who.int/media/docs/default-source/immunization/coverage/who-immuniz.pdf?sfvrsn=72fd7237_2&download=true.

[82] Akhatova A., Azizan A., Atageldiyeva K., Ashimkhanova A., Marat A., Iztleuov Y., Suleimenova A., Shamkeeva S., Aimagambetova G. (2022). Prophylactic Human Papillomavirus Vaccination: From the Origin to the Current State. Vaccines.

[83] Dorji T., Tshomo U., Gyamtsho S., Tamang S.T., Wangmo S., Pongpirul K. (2022). Gender-neutral HPV elimination, cervical cancer screening, and treatment: Experience from Bhutan. Int. J. Gynecol. Obstet..

[84] Dykens J.A., Peterson C.E., Holt H.K., Harper D.M. (2023). Gender neutral HPV vaccination programs: Reconsidering policies to expand cancer prevention globally. Front. Public Health.

[85] Amponsah-Dacosta E., Blose N., Nkwinika V.V., Chepkurui V. (2022). Human Papillomavirus Vaccination in South Africa: Programmatic Challenges and Opportunities for Integration With Other Adolescent Health Services?. Front. Public Health.

[86] Chido-Amajuoyi O.G., Fokom Domgue J., Obi-Jeff C., Schmeler K., Shete S. (2019). A call for the introduction of gender-neutral HPV vaccination to national immunisation programmes in Africa. Lancet Glob. Health.

[87] Ma Y., Wang C., Liu F., Lian G., Li S., He Q., Li T. (2021). Human papillomavirus vaccination coverage and knowledge, perceptions and influencing factors among university students in Guangzhou, China. Hum. Vaccin. Immunother..

[88] Simms K.T., Hanley S.J.B., Smith M.A., Keane A., Canfell K. (2020). Impact of HPV vaccine hesitancy on cervical cancer in Japan: A modelling study. Lancet Public Health.

[89] Nogueira-Rodrigues A., Flores M.G., Macedo Neto A.O., Braga L.A.C., Vieira C.M., de Sousa-Lima R.M., de Andrade D.A.P., Machado K.K., Guimarães A.P.G. (2022). HPV vaccination in Latin America: Coverage status, implementation challenges and strategies to overcome it. Front. Oncol..

[90] Athanasiou A., Bowden S., Paraskevaidi M., Fotopoulou C., Martin-Hirsch P., Paraskevaidis E., Kyrgiou M. (2020). HPV vaccination and cancer prevention. Best Pract. Res. Clin. Obstet. Gynaecol..

[91] Sheikh S., Biundo E., Courcier S., Damm O., Launay O., Maes E., Marcos C., Matthews S., Meijer C., Poscia A. (2018). A report on the status of vaccination in Europe. Vaccine.

[92] Nguyen-Huu N.H., Thilly N., Derrough T., Sdona E., Claudot F., Pulcini C., Agrinier N., HPV Policy working group (2020). Human papillomavirus vaccination coverage, policies, and practical implementation across Europe. Vaccine.

[93] Italian Communication Campaign on HPV Vaccination. https://www.salute.gov.it/portale/vaccinazioni/dettaglioCampagneVaccinazioni.jsp?lingua=italiano&.

[94] Giuliano A.R., Nyitray A.G., Albero G. (2011). Male circumcision and HPV transmission to female partners. Lancet.

[95] (2020). Global Strategy to Accelerate the Elimination of Cervical Cancer as a Public Health Problem. Geneva: World Health Organization. https://www.who.int/publications/i/item/9789240014107.

[96] Sami J., Lemoupa Makajio S., Jeannot E., Kenfack B., Viñals R., Vassilakos P., Petignat P. (2022). Smartphone-Based Visual Inspection with Acetic Acid: An Innovative Tool to Improve Cervical Cancer Screening in Low-Resource Setting. Healthcare.

[97] Khairkhah N., Bolhassani A., Najafipour R. (2022). Current and future direction in treatment of HPV-related cervical disease. J. Mol. Med..

[98] Cooper D.B., Dunton C.J. (2023). Colposcopy, 16 July 2022. StatPearls.

[99] Bhatla N., Singhal S. (2020). Primary HPV screening for cervical cancer. Best Pract. Res. Clin. Obstet. Gynaecol..

[100] Tota J.E., Bentley J., Blake J., Coutlée F., Duggan M.A., Ferenczy A., Franco E.L., Fung-Kee-Fung M., Gotlieb W., Mayrand M.-H. (2017). Introduction of molecular HPV testing as the primary technology in cervical cancer screening: Acting on evidence to change the current paradigm. Prev. Med..

[101] Ebisch R.M.F., Rijstenberg L.L., Soltani G.G., van der Horst J., Vedder J.E.M., Hermsen M., Bosgraaf R.P., Massuger L.F.A.G., Meijer C.J.L.M., Heideman D.A.M. (2022). Adjunctive use of p16 immunohistochemistry for optimizing management of CIN lesions in a high-risk human papillomavirus-positive population. Acta Obstet. Gynecol. Scand..

[102] Stoler M.H., Wright T.C., Ferenczy A., Ranger-Moore J., Fang Q., Kapadia M., Ridder R. (2018). Routine Use of Adjunctive p16 Immunohistochemistry Improves Diagnostic Agreement of Cervical Biopsy Interpretation: Results From the CERTAIN Study. Am. J. Surg. Pathol..

[103] Serrano B., Ibáñez R., Robles C., Peremiquel-Trillas P., de Sanjosé S., Bruni L. (2022). Worldwide use of HPV self-sampling for cervical cancer screening. Prev. Med..

[104] Nishimura H., Yeh P.T., Oguntade H., Kennedy C.E., Narasimhan M. (2021). HPV self-sampling for cervical cancer screening: A systematic review of values and preferences. BMJ Glob. Health.

[105] Profozić Z., Meštrović T., Savić I., Profozić V. (2016). Prevalence of HPV Infection in Croatian Men during a 12-year Period: A Comparative Study of External Genital and Urethral Swabs. Cent. Eur. J. Public. Health.

[106] Pan L.J., Ma J.H., Zhang F.L., Pan F., Zhao D., Zhang X.Y. (2018). HPV infection of the external genitalia in men whose female partners have cervical HPV infection. Zhonghua Nan Ke Xue.

[107] Luttmer R., Dijkstra M.G., Snijders P.J.F., Jordanova E.S., King A.J., Pronk D.T., Meijer C.J., Heideman D.A.M., Doorbar J., Bleeker M.C.G. (2015). Presence of human papillomavirus in semen of healthy men is firmly associated with HPV infections of the penile epithelium. Fertil. Steril..

[108] Tuan L.A., Prem K., Pham Q.D., Toh Z.Q., Tran H.P., Nguyen P.D., Mai C.T.N., Ly L.T.K., Cao V., Le-Ha T.-D. (2021). Anal human papillomavirus prevalence and risk factors among men who have sex with men in Vietnam. Int. J. Infect. Dis..

[109] Shapiro G.K. (2022). HPV Vaccination: An Underused Strategy for the Prevention of Cancer. Curr. Oncol..

[110] Kisling L.A., M Das J. (2022). Prevention Strategies, 2022 May 8. StatPearls.

[111] Swedish K.A., Factor S.H., Goldstone S.E. (2012). Prevention of recurrent high-grade anal neoplasia with quadrivalent human papillomavirus vaccination of men who have sex with men: A nonconcurrent cohort study. Clin. Infect. Dis..

[112] Deshmukh A.A., Cantor S.B., Fenwick E., Chiao E.Y., Nyitray A.G., Stier E.A., Goldstone S.E., Wilkin T., Chhatwal J. (2017). Adjuvant HPV vaccination for anal cancer prevention in HIV-positive men who have sex with men: The time is now. Vaccine.

[113] Ghelardi A., Parazzini F., Martella F., Pieralli A., Bay P., Tonetti A., Svelato A., Bertacca G., Lombardi S., Joura E.A. (2018). SPERANZA project: HPV vaccination after treatment for CIN2. Gynecol. Oncol..

[114] Michalczyk K., Misiek M., Chudecka-Głaz A. (2022). Can Adjuvant HPV Vaccination Be Helpful in the Prevention of Persistent/Recurrent Cervical Dysplasia after Surgical Treatment?—A Literature Review. Cancers.

[115] Di Donato V., Caruso G., Bogani G., Cavallari E.N., Palaia G., Perniola G., Ralli M., Sorrenti S., Romeo U., Pernazza A. (2022). HPV Vaccination after Primary Treatment of HPV-Related Disease across Different Organ Sites: A Multidisciplinary Comprehensive Review and Meta-Analysis. Vaccines.

[116] Kin Cho Goon P., Scholtz L.U., Sudhoff H. (2017). Recurrent respiratory papillomatosis (RRP)-time for a reckoning?. Laryngoscope Investig. Otolaryngol..

[117] Swedish K.A., Goldstone S.E. (2014). Prevention of anal condyloma with quadrivalent human papillomavirus vaccination of older men who have sex with men. PLoS ONE.

[118] Goon P., Sauzet O., Schuermann M., Oppel F., Shao S., Scholtz L.U., Sudhoff H., Goerner M. (2023). Recurrent Respiratory Papillomatosis (RRP)-Meta-analyses on the use of the HPV vaccine as adjuvant therapy. NPJ Vaccines.

[119] Husein-ElAhmed H. (2020). Could the human papillomavirus vaccine prevent recurrence of ano-genital warts?: A systematic review and meta-analysis. Int. J. STD AIDS.

[120] Mirghani H., Jung A.C., Fakhry C. (2017). Primary, secondary and tertiary prevention of human papillomavirus-driven head and neck cancers. Eur. J. Cancer.

[121] Rettig E.M., Wentz A., Posner M.R., Gross N.D., Haddad R.I., Gillison M.L., Fakhry C., Quon H., Sikora A.G., Stott W.J. (2015). Prognostic implication of persistent human papillomavirus type 16 DNA detection in oral rinses for human papillomavirus-related oropharyngeal carcinoma. JAMA Oncol..

[122] Fakhry C., Qualliotine J.R., Zhang Z., Agrawal N., Gaykalova D.A., Bishop J.A., Subramaniam R.M., Koch W.M., Chung C.H., Eisele D.W. (2016). Serum antibodies to HPV16 early proteins warrant investigation as potential biomarkers for risk stratifica- tion and recurrence of HPV-associated oropharyngeal cancer. Cancer Prev. Res..

[123] Dahlstrom K.R., Li G., Hussey C.S., Vo J.T., Wei Q., Zhao C., Sturgis E.M. (2015). Circulating human papillomavirus DNA as a marker for disease extent and recurrence among patients with oropharyngeal cancer. Cancer.

[124] Lin J.C., Wang W.Y., Chen K.Y., Wei Y.H., Liang W.M., Jan J.S., Jiang R.-S. (2004). Quantification of plasma Epstein-Barr virus DNA in patients with advanced nasopharyngeal carcinoma. N. Engl. J. Med..

[125] Twu C.W., Wang W.Y., Liang W.M., Jan J.S., Jiang R.S., Chao J., Jin Y.T., Lin J.C. (2007). Comparison of the prognostic impact of serum anti-EBV anti- body and plasma EBV DNA assays in nasopharyngeal carcinoma. Int. J. Radiat. Oncol. Biol. Phys..

[126] Chrysostomou A.C., Stylianou D.C., Constantinidou A., Kostrikis L.G. (2018). Cervical Cancer Screening Programs in Europe: The Transition Towards HPV Vaccination and Population-Based HPV Testing. Viruses.

[127] Bosco R., Messina G., Aiello B., Guarducci G., Nante N. (2023). The Structures and Activities of Health Promotion in the Italian NHS. Healthcare.

[128] Palfrey S. (2016). New initiatives to improve HPV vaccination rates. Hum. Vaccin. Immunother..

